# Diferrocen­yl(meth­yl)phenyl­silane

**DOI:** 10.1107/S1600536811014917

**Published:** 2011-04-29

**Authors:** Yu-Peng Liu, Zong-Qi Li, Yong-Xia Tan, Zhi-Jie Zhang

**Affiliations:** a Beijing National Laboratory for Molecular Sciences (BNLMS), Institute of Chemistry, Chinese Academy of Sciences, Beijing 100190, People’s Republic of China

## Abstract

In the title mol­ecule, [Fe_2_(C_5_H_5_)_2_(C_17_H_16_Si)], the cyclo­penta­dienyl rings linked to the same Fe atom are approximately eclipsed and the inter­planar angles are 1.8 (2) and 3.4 (2)°. The Fe atom is slightly closer to the substituted cyclo­penta­dienyl ring.

## Related literature

For general background, see: Togni & Hayashi (1994 ▶). 
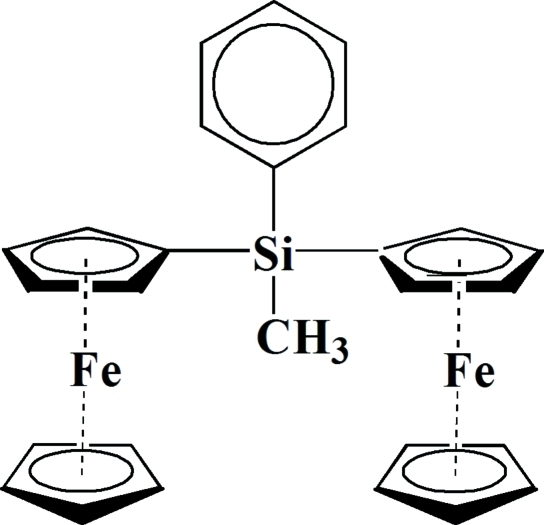


## Experimental

### 

#### Crystal data

[Fe_2_(C_5_H_5_)_2_(C_17_H_16_Si)]
*M* *_r_* = 490.27
Triclinic, 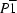

*a* = 7.3840 (15) Å
*b* = 11.749 (2) Å
*c* = 13.107 (3) Å
α = 84.18 (3)°
β = 76.18 (3)°
γ = 79.04 (3)°
*V* = 1082.2 (4) Å^3^
*Z* = 2
Mo *K*α radiation
μ = 1.41 mm^−1^
*T* = 173 K
0.30 × 0.28 × 0.07 mm

#### Data collection

Rigaku MM007HF + CCD (Saturn724+) diffractometer
Absorption correction: multi-scan (*CrystalClear*; Rigaku, 2008 ▶) *T*_min_ = 0.368, *T*_max_ = 1.000
14130 measured reflections
4942 independent reflections
4477 reflections with *I* > 2σ(*I*)
*R*_int_ = 0.070

#### Refinement

*R*[*F* ^2^ > 2σ(*F* ^2^)] = 0.050
*wR*(*F* ^2^) = 0.134
*S* = 1.13
4942 reflections
271 parameters
H-atom parameters constrained
Δρ_max_ = 0.57 e Å^−3^
Δρ_min_ = −0.66 e Å^−3^

### 

Data collection: *CrystalClear* (Rigaku, 2008 ▶); cell refinement: *CrystalClear*; data reduction: *CrystalClear*; program(s) used to solve structure: *SHELXS97* (Sheldrick, 2008 ▶); program(s) used to refine structure: *SHELXL97* (Sheldrick, 2008 ▶); molecular graphics: *Mercury* (Macrae *et al.*, 2008 ▶); software used to prepare material for publication: *SHELXL97*.

## Supplementary Material

Crystal structure: contains datablocks global, I. DOI: 10.1107/S1600536811014917/qm2006sup1.cif

Structure factors: contains datablocks I. DOI: 10.1107/S1600536811014917/qm2006Isup2.hkl

Additional supplementary materials:  crystallographic information; 3D view; checkCIF report

## Figures and Tables

**Fig. 1.**
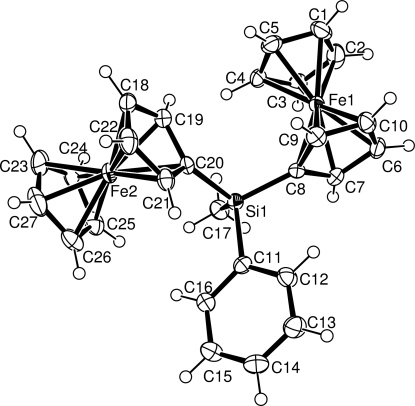
The structure of the title compound, showing 50% probability displacement ellipsoids and the atom-numbering scheme.

**Fig. 2.**
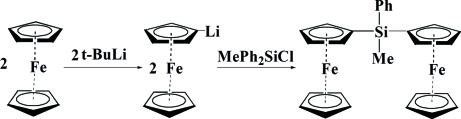
Reaction scheme for synthesis of methylphenyldiferrocenylsilane

## supplementary crystallographic information

### Comment

Ferrocene compounds are of great interest in the field of material chemistry (Togni & Hayashi, 1994). In this paper we reported the synthesis and crystal structure of the title compound. The molecular structure of the compound consists of a methylphenylsilyl unit and two ferrocene units. In the ferrocene unit, the cyclopentaienyl rings linked to the same Fe atom are approximately eclipsed and the interplanar angles are 1.76 (24) and 3.35 (21)°, respectively. The Fe atom is slightly closer to the substituted cyclopentadienyl ring. The distances of the Fe1 atom from the centroids of the unsubstituted and substituted cyclopentadienyl (Cp) rings is 1.648 (1) and 1.645 (1) Å, respectively. The distances of the Fe2 atom from the centroids of the unsubstituted and substituted Cp rings is 1.653 (2) and 1.650 (2) Å, respectively.

### Experimental

Ferrocene (2.00 g, 26.88 mmol) was dissolved in 12 ml of anhydrous tetrahydrofuran (THF). In the course of 15 min a solution of 10.8 mmol *t*-BuLi (7.16 ml of a 1.5 *M n*-Pentance solution) was added dropwise at 0°C. *n*-Hexane (16 ml) was then added, and the solution was kept at -78°C for 15 min, before the orange precipitate was filtered off. The precipitate was washed with small portions of *n*-hexane. The FcLi was dissolved in THF (15 ml) and added to a solution of dichloromethylphenylsilane (0.8 g, 4.69 mmol) in n-hexane (20 ml) at 0°C, and then stirred overnight at room temperature. The precipitate was filtered and the solvent was evaporated under vacuum. the orange residue was purified by recrystallization from *n*-hexane to give 4.04 g of orange product in 88% yield.

### Refinement

All the H atoms were discernible in the difference electron density maps. Nevertheless, all the H atoms were constrained by the riding-hydrogen formalism with *U*_iso_(H) = 1.2*U*_eq_(C_aryl_ or _cyclopentadienyl_) or *U*_iso_(H) = 1.5*U*_eq_(C_methyl_). The C—H distances were constrained to 0.95 Å for the aryl H atoms, 0.98 Å for the the methyl H atoms and 1.00 Å for the cyclopentadienyl H atoms respectively.

### Crystal data

**Table:**

[Fe_2_(C_5_H_5_)_2_(C_17_H_16_Si)]	*Z* = 2
*M**_r_* = 490.27	*F*(000) = 508
Triclinic, *P*1	*D*_x_ = 1.505 Mg m^−^^3^
*a* = 7.3840 (15) Å	Mo *K*α radiation, λ = 0.71073 Å
*b* = 11.749 (2) Å	Cell parameters from 4135 reflections
*c* = 13.107 (3) Å	θ = 1.8–27.5°
α = 84.18 (3)°	µ = 1.41 mm^−^^1^
β = 76.18 (3)°	*T* = 173 K
γ = 79.04 (3)°	Plate, yellow
*V* = 1082.2 (4) Å^3^	0.30 × 0.28 × 0.07 mm

### Data collection

**Table:**

Rigaku MM007HF + CCD (Saturn724+) diffractometer	4942 independent reflections
Radiation source: Rotating Anode	4477 reflections with *I* > 2σ(*I*)
Confocal	*R*_int_ = 0.070
Detector resolution: 28.5714 pixels mm^-1^	θ_max_ = 27.5°, θ_min_ = 1.6°
ω scans at fixed χ = 45°	*h* = −9→9
Absorption correction: multi-scan (*CrystalClear*; Rigaku, 2008)	*k* = −15→15
*T*_min_ = 0.368, *T*_max_ = 1.000	*l* = −16→16
14130 measured reflections	

### Refinement

**Table:**

Refinement on *F*^2^	Primary atom site location: structure-invariant direct methods
Least-squares matrix: full	Secondary atom site location: difference Fourier map
*R*[*F*^2^ > 2σ(*F*^2^)] = 0.050	Hydrogen site location: inferred from neighbouring sites
*wR*(*F*^2^) = 0.134	H-atom parameters constrained
*S* = 1.13	*w* = 1/[σ^2^(*F*_o_^2^) + (0.0607*P*)^2^ + 0.3984*P*] where *P* = (*F*_o_^2^ + 2*F*_c_^2^)/3
4942 reflections	(Δ/σ)_max_ = 0.001
271 parameters	Δρ_max_ = 0.57 e Å^−^^3^
0 restraints	Δρ_min_ = −0.66 e Å^−^^3^

### Special details

**Table:**

Geometry. All e.s.d.'s (except the e.s.d. in the dihedral angle between two l.s. planes) are estimated using the full covariance matrix. The cell e.s.d.'s are taken into account individually in the estimation of e.s.d.'s in distances, angles and torsion angles; correlations between e.s.d.'s in cell parameters are only used when they are defined by crystal symmetry. An approximate (isotropic) treatment of cell e.s.d.'s is used for estimating e.s.d.'s involving l.s. planes.
Refinement. Refinement of *F*^2^ against ALL reflections. The weighted *R*-factor *wR* and goodness of fit *S* are based on *F*^2^, conventional *R*-factors *R* are based on *F*, with *F* set to zero for negative *F*^2^. The threshold expression of *F*^2^ > σ(*F*^2^) is used only for calculating *R*-factors(gt) *etc*. and is not relevant to the choice of reflections for refinement. *R*-factors based on *F*^2^ are statistically about twice as large as those based on *F*, and *R*-factors based on ALL data will be even larger.

### Fractional atomic coordinates and isotropic or equivalent isotropic displacement parameters (Å^2^)

**Table:**

	*x*	*y*	*z*	*U*_iso_*/*U*_eq_	
Fe1	0.53139 (5)	1.10573 (3)	0.67907 (3)	0.02155 (13)	
Fe2	0.97462 (5)	0.61614 (3)	0.69033 (3)	0.02344 (13)	
Si1	0.66477 (10)	0.84806 (6)	0.82468 (6)	0.02047 (17)	
C1	0.5730 (4)	1.1851 (3)	0.5316 (3)	0.0417 (8)	
H1A	0.4763	1.2073	0.4879	0.050*	
C2	0.6085 (4)	1.2555 (3)	0.6041 (3)	0.0411 (8)	
H2A	0.5402	1.3359	0.6205	0.049*	
C3	0.7565 (4)	1.1923 (3)	0.6502 (3)	0.0349 (7)	
H3A	0.8108	1.2200	0.7042	0.042*	
C4	0.8125 (4)	1.0827 (3)	0.6053 (2)	0.0269 (6)	
H4A	0.9128	1.0190	0.6232	0.032*	
C5	0.6997 (4)	1.0771 (3)	0.5324 (2)	0.0323 (7)	
H5A	0.7079	1.0098	0.4895	0.039*	
C6	0.2971 (4)	1.1552 (3)	0.7950 (2)	0.0295 (6)	
H6A	0.2382	1.2368	0.8128	0.035*	
C7	0.4427 (4)	1.0834 (2)	0.8384 (2)	0.0256 (6)	
H7A	0.5039	1.1068	0.8914	0.031*	
C8	0.4895 (4)	0.9728 (2)	0.7921 (2)	0.0210 (5)	
C9	0.3686 (4)	0.9785 (3)	0.7198 (2)	0.0269 (6)	
H9A	0.3685	0.9151	0.6744	0.032*	
C10	0.2520 (4)	1.0901 (3)	0.7216 (2)	0.0292 (6)	
H10A	0.1558	1.1180	0.6784	0.035*	
C11	0.5340 (4)	0.7643 (2)	0.9397 (2)	0.0224 (5)	
C12	0.3377 (4)	0.7909 (3)	0.9725 (2)	0.0289 (6)	
H12A	0.2696	0.8506	0.9348	0.035*	
C13	0.2390 (4)	0.7319 (3)	1.0591 (2)	0.0343 (7)	
H13A	0.1052	0.7519	1.0806	0.041*	
C14	0.3368 (4)	0.6443 (3)	1.1137 (2)	0.0342 (7)	
H14A	0.2697	0.6039	1.1728	0.041*	
C15	0.5298 (4)	0.6153 (3)	1.0833 (2)	0.0326 (7)	
H15A	0.5965	0.5546	1.1207	0.039*	
C16	0.6277 (4)	0.6756 (3)	0.9969 (2)	0.0294 (6)	
H16A	0.7616	0.6558	0.9767	0.035*	
C17	0.8620 (4)	0.9033 (3)	0.8610 (2)	0.0282 (6)	
H17A	0.9279	0.9468	0.8002	0.042*	
H17B	0.9513	0.8376	0.8823	0.042*	
H17C	0.8097	0.9545	0.9195	0.042*	
C18	0.8860 (4)	0.6662 (3)	0.5540 (2)	0.0322 (7)	
H18A	0.9643	0.6534	0.4812	0.039*	
C19	0.8717 (4)	0.7638 (3)	0.6125 (2)	0.0275 (6)	
H19A	0.9387	0.8313	0.5877	0.033*	
C20	0.7460 (3)	0.7498 (2)	0.7137 (2)	0.0217 (5)	
C21	0.6855 (4)	0.6405 (3)	0.7140 (2)	0.0261 (6)	
H21A	0.5992	0.6050	0.7744	0.031*	
C22	0.7713 (4)	0.5892 (3)	0.6167 (2)	0.0339 (7)	
H22A	0.7543	0.5128	0.5961	0.041*	
C23	1.2496 (5)	0.5377 (4)	0.6434 (3)	0.0498 (10)	
H23A	1.3216	0.5273	0.5690	0.060*	
C24	1.2389 (4)	0.6333 (3)	0.7033 (3)	0.0433 (9)	
H24A	1.3012	0.7026	0.6784	0.052*	
C25	1.1252 (4)	0.6138 (3)	0.8041 (3)	0.0336 (7)	
H25A	1.0920	0.6673	0.8629	0.040*	
C26	1.0627 (4)	0.5068 (3)	0.8071 (3)	0.0355 (7)	
H26A	0.9797	0.4705	0.8685	0.043*	
C27	1.1412 (5)	0.4595 (3)	0.7070 (3)	0.0478 (9)	
H27A	1.1231	0.3838	0.6857	0.057*	

### Atomic displacement parameters (Å^2^)

**Table:**

	*U*^11^	*U*^22^	*U*^33^	*U*^12^	*U*^13^	*U*^23^
Fe1	0.0184 (2)	0.0211 (2)	0.0234 (2)	−0.00145 (15)	−0.00342 (15)	−0.00001 (16)
Fe2	0.0189 (2)	0.0215 (2)	0.0292 (2)	0.00110 (15)	−0.00708 (16)	−0.00232 (17)
Si1	0.0193 (3)	0.0208 (4)	0.0208 (4)	−0.0018 (3)	−0.0046 (3)	−0.0016 (3)
C1	0.0285 (15)	0.051 (2)	0.0364 (17)	−0.0002 (14)	−0.0047 (13)	0.0214 (16)
C2	0.0305 (15)	0.0260 (16)	0.056 (2)	−0.0030 (12)	0.0065 (14)	0.0071 (15)
C3	0.0261 (14)	0.0314 (16)	0.0451 (18)	−0.0107 (12)	0.0014 (12)	−0.0037 (14)
C4	0.0189 (12)	0.0289 (15)	0.0282 (14)	−0.0019 (11)	0.0006 (10)	0.0013 (12)
C5	0.0298 (14)	0.0409 (18)	0.0233 (14)	−0.0075 (13)	0.0000 (11)	−0.0006 (13)
C6	0.0212 (13)	0.0282 (15)	0.0326 (15)	0.0015 (11)	0.0024 (11)	−0.0032 (12)
C7	0.0255 (13)	0.0271 (15)	0.0213 (13)	−0.0006 (11)	−0.0012 (10)	−0.0049 (11)
C8	0.0222 (12)	0.0202 (13)	0.0186 (12)	−0.0027 (10)	−0.0023 (9)	0.0020 (10)
C9	0.0239 (13)	0.0270 (15)	0.0310 (14)	−0.0081 (11)	−0.0066 (11)	0.0006 (12)
C10	0.0199 (13)	0.0305 (16)	0.0359 (16)	−0.0043 (11)	−0.0061 (11)	0.0036 (13)
C11	0.0237 (12)	0.0246 (14)	0.0200 (12)	−0.0053 (10)	−0.0047 (10)	−0.0045 (10)
C12	0.0239 (13)	0.0319 (16)	0.0311 (15)	−0.0043 (12)	−0.0093 (11)	0.0041 (13)
C13	0.0247 (14)	0.0423 (19)	0.0353 (16)	−0.0099 (13)	−0.0047 (12)	0.0029 (14)
C14	0.0380 (16)	0.0359 (18)	0.0284 (15)	−0.0141 (14)	−0.0040 (12)	0.0052 (13)
C15	0.0364 (16)	0.0303 (16)	0.0314 (16)	−0.0065 (13)	−0.0108 (12)	0.0059 (13)
C16	0.0277 (14)	0.0282 (15)	0.0288 (15)	−0.0004 (12)	−0.0047 (11)	0.0019 (12)
C17	0.0269 (13)	0.0279 (15)	0.0324 (15)	−0.0060 (11)	−0.0115 (11)	−0.0003 (12)
C18	0.0308 (15)	0.0359 (17)	0.0261 (14)	0.0099 (12)	−0.0091 (12)	−0.0074 (13)
C19	0.0260 (13)	0.0272 (15)	0.0253 (14)	0.0027 (11)	−0.0040 (11)	−0.0008 (12)
C20	0.0189 (12)	0.0208 (13)	0.0232 (13)	0.0034 (10)	−0.0058 (10)	−0.0015 (11)
C21	0.0192 (12)	0.0268 (15)	0.0329 (15)	0.0000 (10)	−0.0094 (11)	−0.0034 (12)
C22	0.0293 (15)	0.0323 (17)	0.0440 (18)	0.0034 (12)	−0.0180 (13)	−0.0132 (14)
C23	0.0258 (16)	0.064 (3)	0.048 (2)	0.0227 (16)	−0.0088 (14)	−0.0053 (19)
C24	0.0211 (14)	0.047 (2)	0.061 (2)	−0.0056 (13)	−0.0126 (14)	0.0098 (18)
C25	0.0264 (14)	0.0362 (18)	0.0411 (17)	0.0021 (12)	−0.0184 (12)	−0.0039 (14)
C26	0.0310 (15)	0.0339 (18)	0.0435 (18)	−0.0026 (13)	−0.0188 (13)	0.0090 (14)
C27	0.055 (2)	0.0306 (18)	0.060 (2)	0.0145 (16)	−0.0331 (19)	−0.0101 (17)

### Geometric parameters (Å, °)

**Table:**

Fe1—C2	2.037 (3)	C7—H7A	1.0000
Fe1—C7	2.039 (3)	C8—C9	1.438 (4)
Fe1—C1	2.040 (3)	C9—C10	1.424 (4)
Fe1—C10	2.043 (3)	C9—H9A	1.0000
Fe1—C9	2.044 (3)	C10—H10A	1.0000
Fe1—C6	2.045 (3)	C11—C16	1.393 (4)
Fe1—C5	2.046 (3)	C11—C12	1.393 (4)
Fe1—C4	2.049 (3)	C12—C13	1.392 (4)
Fe1—C3	2.050 (3)	C12—H12A	0.9500
Fe1—C8	2.055 (3)	C13—C14	1.381 (4)
Fe2—C27	2.033 (3)	C13—H13A	0.9500
Fe2—C19	2.034 (3)	C14—C15	1.370 (4)
Fe2—C23	2.039 (3)	C14—H14A	0.9500
Fe2—C18	2.041 (3)	C15—C16	1.395 (4)
Fe2—C26	2.043 (3)	C15—H15A	0.9500
Fe2—C24	2.047 (3)	C16—H16A	0.9500
Fe2—C21	2.050 (3)	C17—H17A	0.9800
Fe2—C22	2.055 (3)	C17—H17B	0.9800
Fe2—C25	2.057 (3)	C17—H17C	0.9800
Fe2—C20	2.062 (3)	C18—C19	1.416 (4)
Si1—C8	1.850 (3)	C18—C22	1.422 (5)
Si1—C20	1.862 (3)	C18—H18A	1.0000
Si1—C17	1.877 (3)	C19—C20	1.440 (4)
Si1—C11	1.881 (3)	C19—H19A	1.0000
C1—C2	1.420 (5)	C20—C21	1.437 (4)
C1—C5	1.427 (5)	C21—C22	1.420 (4)
C1—H1A	1.0000	C21—H21A	1.0000
C2—C3	1.424 (5)	C22—H22A	1.0000
C2—H2A	1.0000	C23—C27	1.409 (6)
C3—C4	1.418 (4)	C23—C24	1.414 (6)
C3—H3A	1.0000	C23—H23A	1.0000
C4—C5	1.423 (4)	C24—C25	1.409 (5)
C4—H4A	1.0000	C24—H24A	1.0000
C5—H5A	1.0000	C25—C26	1.413 (5)
C6—C10	1.421 (4)	C25—H25A	1.0000
C6—C7	1.428 (4)	C26—C27	1.424 (5)
C6—H6A	1.0000	C26—H26A	1.0000
C7—C8	1.436 (4)	C27—H27A	1.0000
			
C2—Fe1—C7	123.60 (14)	C4—C5—C1	107.5 (3)
C2—Fe1—C1	40.77 (15)	C4—C5—Fe1	69.79 (16)
C7—Fe1—C1	160.03 (14)	C1—C5—Fe1	69.34 (18)
C2—Fe1—C10	119.94 (13)	C4—C5—H5A	126.3
C7—Fe1—C10	68.60 (12)	C1—C5—H5A	126.3
C1—Fe1—C10	106.41 (13)	Fe1—C5—H5A	126.3
C2—Fe1—C9	156.01 (13)	C10—C6—C7	107.7 (3)
C7—Fe1—C9	68.49 (12)	C10—C6—Fe1	69.59 (16)
C1—Fe1—C9	121.19 (14)	C7—C6—Fe1	69.31 (15)
C10—Fe1—C9	40.78 (12)	C10—C6—H6A	126.2
C2—Fe1—C6	105.97 (13)	C7—C6—H6A	126.2
C7—Fe1—C6	40.94 (11)	Fe1—C6—H6A	126.2
C1—Fe1—C6	122.81 (13)	C6—C7—C8	109.1 (3)
C10—Fe1—C6	40.69 (12)	C6—C7—Fe1	69.74 (16)
C9—Fe1—C6	68.58 (12)	C8—C7—Fe1	70.07 (15)
C2—Fe1—C5	68.58 (14)	C6—C7—H7A	125.5
C7—Fe1—C5	157.69 (12)	C8—C7—H7A	125.5
C1—Fe1—C5	40.89 (13)	Fe1—C7—H7A	125.5
C10—Fe1—C5	124.31 (13)	C7—C8—C9	106.2 (2)
C9—Fe1—C5	108.27 (13)	C7—C8—Si1	125.9 (2)
C6—Fe1—C5	160.34 (12)	C9—C8—Si1	127.9 (2)
C2—Fe1—C4	68.10 (13)	C7—C8—Fe1	68.85 (15)
C7—Fe1—C4	122.14 (12)	C9—C8—Fe1	69.06 (15)
C1—Fe1—C4	68.39 (12)	Si1—C8—Fe1	128.41 (13)
C10—Fe1—C4	162.06 (13)	C10—C9—C8	109.0 (3)
C9—Fe1—C4	125.99 (12)	C10—C9—Fe1	69.57 (16)
C6—Fe1—C4	156.61 (13)	C8—C9—Fe1	69.89 (15)
C5—Fe1—C4	40.67 (12)	C10—C9—H9A	125.5
C2—Fe1—C3	40.78 (13)	C8—C9—H9A	125.5
C7—Fe1—C3	107.28 (13)	Fe1—C9—H9A	125.5
C1—Fe1—C3	68.78 (14)	C6—C10—C9	108.1 (2)
C10—Fe1—C3	155.47 (13)	C6—C10—Fe1	69.71 (16)
C9—Fe1—C3	162.23 (13)	C9—C10—Fe1	69.65 (15)
C6—Fe1—C3	120.32 (13)	C6—C10—H10A	125.9
C5—Fe1—C3	68.70 (13)	C9—C10—H10A	125.9
C4—Fe1—C3	40.47 (12)	Fe1—C10—H10A	125.9
C2—Fe1—C8	161.00 (14)	C16—C11—C12	117.2 (2)
C7—Fe1—C8	41.07 (11)	C16—C11—Si1	122.1 (2)
C1—Fe1—C8	157.15 (14)	C12—C11—Si1	120.7 (2)
C10—Fe1—C8	69.26 (11)	C13—C12—C11	121.5 (3)
C9—Fe1—C8	41.05 (10)	C13—C12—H12A	119.2
C6—Fe1—C8	69.36 (11)	C11—C12—H12A	119.2
C5—Fe1—C8	121.88 (12)	C14—C13—C12	119.6 (3)
C4—Fe1—C8	108.47 (11)	C14—C13—H13A	120.2
C3—Fe1—C8	124.50 (13)	C12—C13—H13A	120.2
C27—Fe2—C19	156.38 (14)	C15—C14—C13	120.5 (3)
C27—Fe2—C23	40.47 (16)	C15—C14—H14A	119.8
C19—Fe2—C23	120.54 (14)	C13—C14—H14A	119.8
C27—Fe2—C18	120.80 (14)	C14—C15—C16	119.5 (3)
C19—Fe2—C18	40.67 (12)	C14—C15—H15A	120.2
C23—Fe2—C18	104.90 (14)	C16—C15—H15A	120.2
C27—Fe2—C26	40.89 (15)	C11—C16—C15	121.7 (3)
C19—Fe2—C26	160.52 (13)	C11—C16—H16A	119.1
C23—Fe2—C26	68.35 (15)	C15—C16—H16A	119.1
C18—Fe2—C26	158.35 (14)	Si1—C17—H17A	109.5
C27—Fe2—C24	68.03 (15)	Si1—C17—H17B	109.5
C19—Fe2—C24	106.61 (13)	H17A—C17—H17B	109.5
C23—Fe2—C24	40.48 (16)	Si1—C17—H17C	109.5
C18—Fe2—C24	121.03 (14)	H17A—C17—H17C	109.5
C26—Fe2—C24	68.00 (13)	H17B—C17—H17C	109.5
C27—Fe2—C21	124.21 (15)	C19—C18—C22	108.4 (3)
C19—Fe2—C21	68.25 (12)	C19—C18—Fe2	69.39 (17)
C23—Fe2—C21	158.41 (16)	C22—C18—Fe2	70.21 (17)
C18—Fe2—C21	68.04 (12)	C19—C18—H18A	125.8
C26—Fe2—C21	110.28 (13)	C22—C18—H18A	125.8
C24—Fe2—C21	160.67 (14)	Fe2—C18—H18A	125.8
C27—Fe2—C22	106.88 (14)	C18—C19—C20	109.1 (3)
C19—Fe2—C22	68.52 (13)	C18—C19—Fe2	69.93 (17)
C23—Fe2—C22	121.03 (15)	C20—C19—Fe2	70.49 (15)
C18—Fe2—C22	40.62 (13)	C18—C19—H19A	125.5
C26—Fe2—C22	123.88 (13)	C20—C19—H19A	125.5
C24—Fe2—C22	156.87 (14)	Fe2—C19—H19A	125.5
C21—Fe2—C22	40.48 (12)	C21—C20—C19	105.6 (2)
C27—Fe2—C25	68.00 (14)	C21—C20—Si1	124.2 (2)
C19—Fe2—C25	123.67 (13)	C19—C20—Si1	130.2 (2)
C23—Fe2—C25	67.84 (14)	C21—C20—Fe2	69.11 (15)
C18—Fe2—C25	158.03 (14)	C19—C20—Fe2	68.37 (15)
C26—Fe2—C25	40.31 (13)	Si1—C20—Fe2	127.87 (14)
C24—Fe2—C25	40.16 (13)	C22—C21—C20	109.6 (3)
C21—Fe2—C25	126.03 (12)	C22—C21—Fe2	69.92 (16)
C22—Fe2—C25	160.92 (13)	C20—C21—Fe2	69.99 (15)
C27—Fe2—C20	160.94 (15)	C22—C21—H21A	125.2
C19—Fe2—C20	41.14 (10)	C20—C21—H21A	125.2
C23—Fe2—C20	157.74 (15)	Fe2—C21—H21A	125.2
C18—Fe2—C20	69.06 (11)	C21—C22—C18	107.3 (3)
C26—Fe2—C20	124.83 (13)	C21—C22—Fe2	69.60 (16)
C24—Fe2—C20	123.02 (13)	C18—C22—Fe2	69.17 (17)
C21—Fe2—C20	40.90 (11)	C21—C22—H22A	126.3
C22—Fe2—C20	69.11 (12)	C18—C22—H22A	126.3
C25—Fe2—C20	109.36 (12)	Fe2—C22—H22A	126.3
C8—Si1—C20	109.90 (12)	C27—C23—C24	108.0 (3)
C8—Si1—C17	109.23 (13)	C27—C23—Fe2	69.52 (19)
C20—Si1—C17	113.67 (13)	C24—C23—Fe2	70.07 (19)
C8—Si1—C11	106.11 (12)	C27—C23—H23A	126.0
C20—Si1—C11	106.86 (12)	C24—C23—H23A	126.0
C17—Si1—C11	110.79 (12)	Fe2—C23—H23A	126.0
C2—C1—C5	107.8 (3)	C25—C24—C23	108.2 (3)
C2—C1—Fe1	69.52 (18)	C25—C24—Fe2	70.30 (17)
C5—C1—Fe1	69.77 (16)	C23—C24—Fe2	69.45 (19)
C2—C1—H1A	126.1	C25—C24—H24A	125.9
C5—C1—H1A	126.1	C23—C24—H24A	125.9
Fe1—C1—H1A	126.1	Fe2—C24—H24A	125.9
C1—C2—C3	108.6 (3)	C24—C25—C26	108.3 (3)
C1—C2—Fe1	69.71 (18)	C24—C25—Fe2	69.54 (18)
C3—C2—Fe1	70.10 (17)	C26—C25—Fe2	69.31 (17)
C1—C2—H2A	125.7	C24—C25—H25A	125.8
C3—C2—H2A	125.7	C26—C25—H25A	125.8
Fe1—C2—H2A	125.7	Fe2—C25—H25A	125.8
C4—C3—C2	107.2 (3)	C25—C26—C27	107.5 (3)
C4—C3—Fe1	69.71 (16)	C25—C26—Fe2	70.38 (17)
C2—C3—Fe1	69.12 (17)	C27—C26—Fe2	69.16 (18)
C4—C3—H3A	126.4	C25—C26—H26A	126.3
C2—C3—H3A	126.4	C27—C26—H26A	126.3
Fe1—C3—H3A	126.4	Fe2—C26—H26A	126.3
C3—C4—C5	108.9 (3)	C23—C27—C26	108.1 (3)
C3—C4—Fe1	69.82 (16)	C23—C27—Fe2	70.0 (2)
C5—C4—Fe1	69.54 (16)	C26—C27—Fe2	69.95 (19)
C3—C4—H4A	125.5	C23—C27—H27A	125.9
C5—C4—H4A	125.5	C26—C27—H27A	125.9
Fe1—C4—H4A	125.5	Fe2—C27—H27A	125.9
			
C7—Fe1—C1—C2	44.7 (4)	Si1—C11—C12—C13	−178.2 (2)
C10—Fe1—C1—C2	117.1 (2)	C11—C12—C13—C14	−0.5 (5)
C9—Fe1—C1—C2	158.97 (18)	C12—C13—C14—C15	0.1 (5)
C6—Fe1—C1—C2	75.7 (2)	C13—C14—C15—C16	0.5 (5)
C5—Fe1—C1—C2	−119.0 (3)	C12—C11—C16—C15	0.3 (4)
C4—Fe1—C1—C2	−81.1 (2)	Si1—C11—C16—C15	178.8 (2)
C3—Fe1—C1—C2	−37.47 (19)	C14—C15—C16—C11	−0.7 (5)
C8—Fe1—C1—C2	−167.5 (3)	C27—Fe2—C18—C19	−160.54 (19)
C2—Fe1—C1—C5	119.0 (3)	C23—Fe2—C18—C19	−119.8 (2)
C7—Fe1—C1—C5	163.7 (3)	C26—Fe2—C18—C19	171.5 (3)
C10—Fe1—C1—C5	−123.9 (2)	C24—Fe2—C18—C19	−79.1 (2)
C9—Fe1—C1—C5	−82.0 (2)	C21—Fe2—C18—C19	81.73 (18)
C6—Fe1—C1—C5	−165.25 (18)	C22—Fe2—C18—C19	119.6 (2)
C4—Fe1—C1—C5	37.94 (19)	C25—Fe2—C18—C19	−52.4 (4)
C3—Fe1—C1—C5	81.6 (2)	C20—Fe2—C18—C19	37.64 (16)
C8—Fe1—C1—C5	−48.5 (4)	C27—Fe2—C18—C22	79.9 (2)
C5—C1—C2—C3	0.0 (3)	C19—Fe2—C18—C22	−119.6 (2)
Fe1—C1—C2—C3	59.5 (2)	C23—Fe2—C18—C22	120.6 (2)
C5—C1—C2—Fe1	−59.5 (2)	C26—Fe2—C18—C22	52.0 (4)
C7—Fe1—C2—C1	−163.23 (18)	C24—Fe2—C18—C22	161.27 (19)
C10—Fe1—C2—C1	−80.3 (2)	C21—Fe2—C18—C22	−37.85 (17)
C9—Fe1—C2—C1	−49.0 (4)	C25—Fe2—C18—C22	−171.9 (3)
C6—Fe1—C2—C1	−122.09 (19)	C20—Fe2—C18—C22	−81.94 (18)
C5—Fe1—C2—C1	37.95 (18)	C22—C18—C19—C20	−0.2 (3)
C4—Fe1—C2—C1	81.9 (2)	Fe2—C18—C19—C20	−59.83 (19)
C3—Fe1—C2—C1	119.7 (3)	C22—C18—C19—Fe2	59.6 (2)
C8—Fe1—C2—C1	165.1 (3)	C27—Fe2—C19—C18	45.6 (4)
C7—Fe1—C2—C3	77.0 (2)	C23—Fe2—C19—C18	76.8 (2)
C1—Fe1—C2—C3	−119.7 (3)	C26—Fe2—C19—C18	−170.6 (3)
C10—Fe1—C2—C3	159.98 (19)	C24—Fe2—C19—C18	118.6 (2)
C9—Fe1—C2—C3	−168.8 (3)	C21—Fe2—C19—C18	−81.17 (19)
C6—Fe1—C2—C3	118.2 (2)	C22—Fe2—C19—C18	−37.48 (18)
C5—Fe1—C2—C3	−81.8 (2)	C25—Fe2—C19—C18	159.14 (18)
C4—Fe1—C2—C3	−37.88 (19)	C20—Fe2—C19—C18	−119.9 (2)
C8—Fe1—C2—C3	45.3 (4)	C27—Fe2—C19—C20	165.5 (3)
C1—C2—C3—C4	0.3 (3)	C23—Fe2—C19—C20	−163.31 (19)
Fe1—C2—C3—C4	59.5 (2)	C18—Fe2—C19—C20	119.9 (2)
C1—C2—C3—Fe1	−59.3 (2)	C26—Fe2—C19—C20	−50.7 (4)
C2—Fe1—C3—C4	−118.6 (3)	C24—Fe2—C19—C20	−121.53 (18)
C7—Fe1—C3—C4	119.59 (19)	C21—Fe2—C19—C20	38.72 (16)
C1—Fe1—C3—C4	−81.2 (2)	C22—Fe2—C19—C20	82.42 (18)
C10—Fe1—C3—C4	−164.2 (3)	C25—Fe2—C19—C20	−81.0 (2)
C9—Fe1—C3—C4	46.3 (5)	C18—C19—C20—C21	0.1 (3)
C6—Fe1—C3—C4	162.33 (17)	Fe2—C19—C20—C21	−59.40 (17)
C5—Fe1—C3—C4	−37.13 (18)	C18—C19—C20—Si1	−178.5 (2)
C8—Fe1—C3—C4	77.7 (2)	Fe2—C19—C20—Si1	122.0 (2)
C7—Fe1—C3—C2	−121.8 (2)	C18—C19—C20—Fe2	59.5 (2)
C1—Fe1—C3—C2	37.5 (2)	C8—Si1—C20—C21	−104.2 (2)
C10—Fe1—C3—C2	−45.6 (4)	C17—Si1—C20—C21	133.1 (2)
C9—Fe1—C3—C2	164.9 (4)	C11—Si1—C20—C21	10.5 (2)
C6—Fe1—C3—C2	−79.0 (2)	C8—Si1—C20—C19	74.2 (3)
C5—Fe1—C3—C2	81.5 (2)	C17—Si1—C20—C19	−48.5 (3)
C4—Fe1—C3—C2	118.6 (3)	C11—Si1—C20—C19	−171.1 (2)
C8—Fe1—C3—C2	−163.69 (19)	C8—Si1—C20—Fe2	166.86 (15)
C2—C3—C4—C5	−0.5 (3)	C17—Si1—C20—Fe2	44.1 (2)
Fe1—C3—C4—C5	58.71 (19)	C11—Si1—C20—Fe2	−78.43 (19)
C2—C3—C4—Fe1	−59.2 (2)	C27—Fe2—C20—C21	−44.6 (5)
C2—Fe1—C4—C3	38.2 (2)	C19—Fe2—C20—C21	117.5 (2)
C7—Fe1—C4—C3	−78.7 (2)	C23—Fe2—C20—C21	158.2 (3)
C1—Fe1—C4—C3	82.2 (2)	C18—Fe2—C20—C21	80.22 (18)
C10—Fe1—C4—C3	158.5 (3)	C26—Fe2—C20—C21	−80.9 (2)
C9—Fe1—C4—C3	−164.17 (19)	C24—Fe2—C20—C21	−165.60 (18)
C6—Fe1—C4—C3	−41.3 (4)	C22—Fe2—C20—C21	36.60 (17)
C5—Fe1—C4—C3	120.3 (3)	C25—Fe2—C20—C21	−123.14 (17)
C8—Fe1—C4—C3	−121.91 (19)	C27—Fe2—C20—C19	−162.1 (4)
C2—Fe1—C4—C5	−82.2 (2)	C23—Fe2—C20—C19	40.8 (4)
C7—Fe1—C4—C5	160.96 (18)	C18—Fe2—C20—C19	−37.23 (18)
C1—Fe1—C4—C5	−38.1 (2)	C26—Fe2—C20—C19	161.67 (18)
C10—Fe1—C4—C5	38.1 (4)	C24—Fe2—C20—C19	77.0 (2)
C9—Fe1—C4—C5	75.5 (2)	C21—Fe2—C20—C19	−117.5 (2)
C6—Fe1—C4—C5	−161.7 (3)	C22—Fe2—C20—C19	−80.85 (18)
C3—Fe1—C4—C5	−120.3 (3)	C25—Fe2—C20—C19	119.41 (18)
C8—Fe1—C4—C5	117.75 (19)	C27—Fe2—C20—Si1	73.1 (5)
C3—C4—C5—C1	0.5 (3)	C19—Fe2—C20—Si1	−124.8 (3)
Fe1—C4—C5—C1	59.3 (2)	C23—Fe2—C20—Si1	−84.1 (4)
C3—C4—C5—Fe1	−58.9 (2)	C18—Fe2—C20—Si1	−162.1 (2)
C2—C1—C5—C4	−0.3 (3)	C26—Fe2—C20—Si1	36.8 (2)
Fe1—C1—C5—C4	−59.62 (19)	C24—Fe2—C20—Si1	−47.9 (2)
C2—C1—C5—Fe1	59.3 (2)	C21—Fe2—C20—Si1	117.7 (2)
C2—Fe1—C5—C4	80.9 (2)	C22—Fe2—C20—Si1	154.3 (2)
C7—Fe1—C5—C4	−46.7 (4)	C25—Fe2—C20—Si1	−5.4 (2)
C1—Fe1—C5—C4	118.7 (3)	C19—C20—C21—C22	0.1 (3)
C10—Fe1—C5—C4	−166.69 (17)	Si1—C20—C21—C22	178.83 (19)
C9—Fe1—C5—C4	−124.43 (18)	Fe2—C20—C21—C22	−58.82 (19)
C6—Fe1—C5—C4	158.2 (3)	C19—C20—C21—Fe2	58.92 (17)
C3—Fe1—C5—C4	36.96 (18)	Si1—C20—C21—Fe2	−122.35 (19)
C8—Fe1—C5—C4	−81.3 (2)	C27—Fe2—C21—C22	−75.2 (2)
C2—Fe1—C5—C1	−37.8 (2)	C19—Fe2—C21—C22	81.96 (19)
C7—Fe1—C5—C1	−165.4 (3)	C23—Fe2—C21—C22	−36.6 (4)
C10—Fe1—C5—C1	74.6 (2)	C18—Fe2—C21—C22	37.98 (19)
C9—Fe1—C5—C1	116.9 (2)	C26—Fe2—C21—C22	−118.9 (2)
C6—Fe1—C5—C1	39.5 (5)	C24—Fe2—C21—C22	160.0 (4)
C4—Fe1—C5—C1	−118.7 (3)	C25—Fe2—C21—C22	−161.43 (19)
C3—Fe1—C5—C1	−81.8 (2)	C20—Fe2—C21—C22	120.9 (2)
C8—Fe1—C5—C1	159.98 (19)	C27—Fe2—C21—C20	163.90 (18)
C2—Fe1—C6—C10	117.61 (19)	C19—Fe2—C21—C20	−38.95 (16)
C7—Fe1—C6—C10	−119.1 (2)	C23—Fe2—C21—C20	−157.5 (3)
C1—Fe1—C6—C10	76.4 (2)	C18—Fe2—C21—C20	−82.93 (18)
C9—Fe1—C6—C10	−37.69 (16)	C26—Fe2—C21—C20	120.23 (18)
C5—Fe1—C6—C10	46.8 (4)	C24—Fe2—C21—C20	39.1 (4)
C4—Fe1—C6—C10	−170.8 (3)	C22—Fe2—C21—C20	−120.9 (2)
C3—Fe1—C6—C10	159.45 (17)	C25—Fe2—C21—C20	77.7 (2)
C8—Fe1—C6—C10	−81.81 (18)	C20—C21—C22—C18	−0.3 (3)
C2—Fe1—C6—C7	−123.26 (19)	Fe2—C21—C22—C18	−59.1 (2)
C1—Fe1—C6—C7	−164.42 (19)	C20—C21—C22—Fe2	58.86 (19)
C10—Fe1—C6—C7	119.1 (2)	C19—C18—C22—C21	0.3 (3)
C9—Fe1—C6—C7	81.44 (18)	Fe2—C18—C22—C21	59.39 (19)
C5—Fe1—C6—C7	165.9 (3)	C19—C18—C22—Fe2	−59.1 (2)
C4—Fe1—C6—C7	−51.7 (4)	C27—Fe2—C22—C21	123.3 (2)
C3—Fe1—C6—C7	−81.4 (2)	C19—Fe2—C22—C21	−81.24 (19)
C8—Fe1—C6—C7	37.32 (17)	C23—Fe2—C22—C21	165.2 (2)
C10—C6—C7—C8	0.0 (3)	C18—Fe2—C22—C21	−118.8 (3)
Fe1—C6—C7—C8	−59.21 (18)	C26—Fe2—C22—C21	81.7 (2)
C10—C6—C7—Fe1	59.24 (19)	C24—Fe2—C22—C21	−163.2 (3)
C2—Fe1—C7—C6	74.8 (2)	C25—Fe2—C22—C21	52.0 (4)
C1—Fe1—C7—C6	41.3 (4)	C20—Fe2—C22—C21	−36.96 (17)
C10—Fe1—C7—C6	−37.71 (18)	C27—Fe2—C22—C18	−117.9 (2)
C9—Fe1—C7—C6	−81.68 (19)	C19—Fe2—C22—C18	37.53 (17)
C5—Fe1—C7—C6	−167.5 (3)	C23—Fe2—C22—C18	−76.1 (2)
C4—Fe1—C7—C6	158.43 (18)	C26—Fe2—C22—C18	−159.51 (18)
C3—Fe1—C7—C6	116.63 (19)	C24—Fe2—C22—C18	−44.5 (4)
C8—Fe1—C7—C6	−120.3 (2)	C21—Fe2—C22—C18	118.8 (3)
C2—Fe1—C7—C8	−164.89 (16)	C25—Fe2—C22—C18	170.8 (3)
C1—Fe1—C7—C8	161.6 (3)	C20—Fe2—C22—C18	81.80 (19)
C10—Fe1—C7—C8	82.57 (17)	C19—Fe2—C23—C27	−161.3 (2)
C9—Fe1—C7—C8	38.60 (15)	C18—Fe2—C23—C27	−120.3 (2)
C6—Fe1—C7—C8	120.3 (2)	C26—Fe2—C23—C27	38.0 (2)
C5—Fe1—C7—C8	−47.2 (4)	C24—Fe2—C23—C27	119.0 (3)
C4—Fe1—C7—C8	−81.30 (19)	C21—Fe2—C23—C27	−52.6 (4)
C3—Fe1—C7—C8	−123.10 (17)	C22—Fe2—C23—C27	−79.5 (2)
C6—C7—C8—C9	−0.2 (3)	C25—Fe2—C23—C27	81.6 (2)
Fe1—C7—C8—C9	−59.25 (17)	C20—Fe2—C23—C27	168.7 (3)
C6—C7—C8—Si1	−178.15 (18)	C27—Fe2—C23—C24	−119.0 (3)
Fe1—C7—C8—Si1	122.8 (2)	C19—Fe2—C23—C24	79.6 (2)
C6—C7—C8—Fe1	59.01 (19)	C18—Fe2—C23—C24	120.7 (2)
C20—Si1—C8—C7	−157.8 (2)	C26—Fe2—C23—C24	−81.0 (2)
C17—Si1—C8—C7	−32.4 (3)	C21—Fe2—C23—C24	−171.6 (3)
C11—Si1—C8—C7	87.1 (2)	C22—Fe2—C23—C24	161.5 (2)
C20—Si1—C8—C9	24.8 (3)	C25—Fe2—C23—C24	−37.4 (2)
C17—Si1—C8—C9	150.1 (2)	C20—Fe2—C23—C24	49.7 (4)
C11—Si1—C8—C9	−90.4 (2)	C27—C23—C24—C25	0.5 (4)
C20—Si1—C8—Fe1	−67.5 (2)	Fe2—C23—C24—C25	59.9 (2)
C17—Si1—C8—Fe1	57.9 (2)	C27—C23—C24—Fe2	−59.4 (2)
C11—Si1—C8—Fe1	177.36 (16)	C27—Fe2—C24—C25	−81.5 (2)
C2—Fe1—C8—C7	41.8 (4)	C19—Fe2—C24—C25	122.9 (2)
C1—Fe1—C8—C7	−163.9 (3)	C23—Fe2—C24—C25	−119.2 (3)
C10—Fe1—C8—C7	−80.84 (18)	C18—Fe2—C24—C25	164.85 (19)
C9—Fe1—C8—C7	−117.9 (2)	C26—Fe2—C24—C25	−37.2 (2)
C6—Fe1—C8—C7	−37.21 (16)	C21—Fe2—C24—C25	51.5 (5)
C5—Fe1—C8—C7	160.84 (17)	C22—Fe2—C24—C25	−163.0 (3)
C4—Fe1—C8—C7	118.06 (17)	C20—Fe2—C24—C25	81.0 (2)
C3—Fe1—C8—C7	76.1 (2)	C27—Fe2—C24—C23	37.7 (2)
C2—Fe1—C8—C9	159.7 (3)	C19—Fe2—C24—C23	−117.9 (2)
C7—Fe1—C8—C9	117.9 (2)	C18—Fe2—C24—C23	−76.0 (3)
C1—Fe1—C8—C9	−46.0 (4)	C26—Fe2—C24—C23	82.0 (2)
C10—Fe1—C8—C9	37.06 (17)	C21—Fe2—C24—C23	170.7 (3)
C6—Fe1—C8—C9	80.69 (18)	C22—Fe2—C24—C23	−43.8 (4)
C5—Fe1—C8—C9	−81.26 (19)	C25—Fe2—C24—C23	119.2 (3)
C4—Fe1—C8—C9	−124.04 (17)	C20—Fe2—C24—C23	−159.8 (2)
C3—Fe1—C8—C9	−166.00 (18)	C23—C24—C25—C26	−0.7 (3)
C2—Fe1—C8—Si1	−77.9 (4)	Fe2—C24—C25—C26	58.6 (2)
C7—Fe1—C8—Si1	−119.7 (3)	C23—C24—C25—Fe2	−59.4 (2)
C1—Fe1—C8—Si1	76.4 (4)	C27—Fe2—C25—C24	81.6 (2)
C10—Fe1—C8—Si1	159.5 (2)	C19—Fe2—C25—C24	−75.1 (2)
C9—Fe1—C8—Si1	122.4 (3)	C23—Fe2—C25—C24	37.7 (2)
C6—Fe1—C8—Si1	−156.9 (2)	C18—Fe2—C25—C24	−36.8 (4)
C5—Fe1—C8—Si1	41.1 (2)	C26—Fe2—C25—C24	119.9 (3)
C4—Fe1—C8—Si1	−1.6 (2)	C21—Fe2—C25—C24	−161.3 (2)
C3—Fe1—C8—Si1	−43.6 (2)	C22—Fe2—C25—C24	159.4 (3)
C7—C8—C9—C10	0.4 (3)	C20—Fe2—C25—C24	−118.6 (2)
Si1—C8—C9—C10	178.22 (19)	C27—Fe2—C25—C26	−38.4 (2)
Fe1—C8—C9—C10	−58.75 (19)	C19—Fe2—C25—C26	164.97 (18)
C7—C8—C9—Fe1	59.12 (17)	C23—Fe2—C25—C26	−82.2 (2)
Si1—C8—C9—Fe1	−123.0 (2)	C18—Fe2—C25—C26	−156.7 (3)
C2—Fe1—C9—C10	−43.5 (4)	C24—Fe2—C25—C26	−119.9 (3)
C7—Fe1—C9—C10	81.75 (19)	C21—Fe2—C25—C26	78.8 (2)
C1—Fe1—C9—C10	−78.7 (2)	C22—Fe2—C25—C26	39.5 (5)
C6—Fe1—C9—C10	37.61 (17)	C20—Fe2—C25—C26	121.4 (2)
C5—Fe1—C9—C10	−121.75 (18)	C24—C25—C26—C27	0.7 (3)
C4—Fe1—C9—C10	−163.38 (17)	Fe2—C25—C26—C27	59.5 (2)
C3—Fe1—C9—C10	161.2 (4)	C24—C25—C26—Fe2	−58.8 (2)
C8—Fe1—C9—C10	120.4 (2)	C27—Fe2—C26—C25	118.5 (3)
C2—Fe1—C9—C8	−163.9 (3)	C19—Fe2—C26—C25	−40.3 (4)
C7—Fe1—C9—C8	−38.62 (16)	C23—Fe2—C26—C25	80.8 (2)
C1—Fe1—C9—C8	160.94 (17)	C18—Fe2—C26—C25	156.4 (3)
C10—Fe1—C9—C8	−120.4 (2)	C24—Fe2—C26—C25	37.1 (2)
C6—Fe1—C9—C8	−82.76 (18)	C21—Fe2—C26—C25	−122.27 (19)
C5—Fe1—C9—C8	117.89 (17)	C22—Fe2—C26—C25	−165.50 (18)
C4—Fe1—C9—C8	76.3 (2)	C20—Fe2—C26—C25	−78.7 (2)
C3—Fe1—C9—C8	40.8 (5)	C19—Fe2—C26—C27	−158.8 (3)
C7—C6—C10—C9	0.2 (3)	C23—Fe2—C26—C27	−37.6 (2)
Fe1—C6—C10—C9	59.27 (19)	C18—Fe2—C26—C27	37.9 (4)
C7—C6—C10—Fe1	−59.06 (19)	C24—Fe2—C26—C27	−81.4 (2)
C8—C9—C10—C6	−0.4 (3)	C21—Fe2—C26—C27	119.3 (2)
Fe1—C9—C10—C6	−59.31 (19)	C22—Fe2—C26—C27	76.0 (3)
C8—C9—C10—Fe1	58.94 (18)	C25—Fe2—C26—C27	−118.5 (3)
C2—Fe1—C10—C6	−79.5 (2)	C20—Fe2—C26—C27	162.8 (2)
C7—Fe1—C10—C6	37.94 (17)	C24—C23—C27—C26	0.0 (4)
C1—Fe1—C10—C6	−121.6 (2)	Fe2—C23—C27—C26	−59.8 (2)
C9—Fe1—C10—C6	119.4 (2)	C24—C23—C27—Fe2	59.8 (2)
C5—Fe1—C10—C6	−162.74 (17)	C25—C26—C27—C23	−0.4 (4)
C4—Fe1—C10—C6	168.1 (3)	Fe2—C26—C27—C23	59.8 (2)
C3—Fe1—C10—C6	−46.9 (4)	C25—C26—C27—Fe2	−60.2 (2)
C8—Fe1—C10—C6	82.09 (18)	C19—Fe2—C27—C23	43.5 (4)
C2—Fe1—C10—C9	161.15 (19)	C18—Fe2—C27—C23	76.3 (3)
C7—Fe1—C10—C9	−81.45 (18)	C26—Fe2—C27—C23	−119.0 (3)
C1—Fe1—C10—C9	119.0 (2)	C24—Fe2—C27—C23	−37.7 (2)
C6—Fe1—C10—C9	−119.4 (2)	C21—Fe2—C27—C23	159.3 (2)
C5—Fe1—C10—C9	77.9 (2)	C22—Fe2—C27—C23	118.3 (2)
C4—Fe1—C10—C9	48.7 (4)	C25—Fe2—C27—C23	−81.2 (2)
C3—Fe1—C10—C9	−166.3 (3)	C20—Fe2—C27—C23	−166.9 (3)
C8—Fe1—C10—C9	−37.29 (16)	C19—Fe2—C27—C26	162.5 (3)
C8—Si1—C11—C16	−168.9 (2)	C23—Fe2—C27—C26	119.0 (3)
C20—Si1—C11—C16	73.8 (3)	C18—Fe2—C27—C26	−164.70 (19)
C17—Si1—C11—C16	−50.5 (3)	C24—Fe2—C27—C26	81.3 (2)
C8—Si1—C11—C12	9.5 (3)	C21—Fe2—C27—C26	−81.7 (2)
C20—Si1—C11—C12	−107.7 (2)	C22—Fe2—C27—C26	−122.7 (2)
C17—Si1—C11—C12	128.0 (2)	C25—Fe2—C27—C26	37.84 (19)
C16—C11—C12—C13	0.3 (4)	C20—Fe2—C27—C26	−47.9 (5)
